# Rethinking the Impact and Management of Diabetes in Heart Failure Patients

**DOI:** 10.1007/s11897-023-00633-x

**Published:** 2023-12-04

**Authors:** Katharina Schütt

**Affiliations:** Department of Internal Medicine I (Cardiology), University Hospital, RWTH Aachen University, Pauwelsstraße 30, 52074 Aachen, Germany

**Keywords:** Diabetes, Heart failure, CKD

## Abstract

**Purpose of Review:**

The following overview article summarizes the most important aspects of diagnosis and screening and provides an overview on the current evidence of glucose-lowering and heart failure treatment in patients with diabetes.

**Recent Findings:**

Patients with diabetes exhibit an increased risk to develop heart failure and the presence of both comorbidities has a major impact on the prognosis of these patients. Thus, it is of utmost importance to detect heart failure in patients with diabetes and to screen all patients with heart failure for the presence of diabetes. Moreover, the diagnosis of heart failure in diabetes often requires an adjustment of medical therapy.

**Summary:**

The presence of the 2 comorbidities, heart failure and diabetes, in a given patient which has a major impact on the prognosis and implementation of guideline-directed therapies to reduce cardiovascular risk in this high-risk population is of critical importance.

## Introduction

The presence of diabetes mellitus (DM) represents a significant risk factor for the development of heart failure (HF). Individuals with DM are approximately 2–5 times more likely to develop heart failure at a younger age [1]. Clinical studies have revealed that approximately 30% of patients with DM already exhibit heart failure, and an even greater proportion remains undiagnosed. Among the cases, 28% involved unrecognized heart failure, with about 25% attributed to heart failure with preserved ejection fraction (HFpEF) and approximately 75% linked to heart failure with reduced ejection fraction (HFrEF) [2]. Conversely, the presence of heart failure leads to a metabolic state conducive to diabetes development and is considered a risk factor for DM [3–5]. Around 30–40% of heart failure patients, regardless of their left ventricular ejection fraction (LVEF), have prediabetes or manifest DM [6–8, 9•, 10–12].

The coexistence of DM and heart failure is associated with a poorer prognosis [8, 11, 13–17], resulting in a 50–90% increase in cardiovascular mortality [8, 16–18]. Two studies have shown that heart failure patients with prediabetes or undiagnosed DM have higher mortality and event rates compared to individuals with normal blood sugar levels [9•, 19].

These findings underscore the importance of early identification and consistent treatment for the simultaneous presence of both conditions.

## Diagnosis of HF/DM

Given the fact that patients with diabetes mellitus have approximately a threefold increased risk of developing heart failure [20], it is crucial to consider the possibility of heart failure if typical symptoms (such as shortness of breath, orthopnea, paroxysmal nocturnal dyspnea, reduced exercise tolerance, fatigue, exhaustion, prolonged recovery time after exertion, ankle swelling) or atypical symptoms (including nocturnal cough, wheezing, bloating, loss of appetite, depression, palpitations, dizziness, syncope) are present. The diagnostic process for heart failure does not differ between patients with and without diabetes. Following the ESC algorithm, the initial focus is on obtaining the patient’s medical history and conducting a clinical examination, followed by an electrocardiogram (ECG). Natriuretic peptides like NT-proBNP or BNP should be measured in the next step, and if their levels are elevated, an echocardiography should be performed. Once the diagnosis of heart failure is confirmed, additional tests such as coronary angiography, cardiac magnetic resonance imaging (MRI), or biopsy may be conducted to determine the underlying cause of heart failure [21••].

On the other hand, the prevalence of DM among patients with heart failure ranges from 25 to 40% [22], with a significant number of patients having undiagnosed diabetes. Since the coexistence of both conditions significantly impacts prognosis, it is important to actively screen all patients with heart failure for the presence of diabetes. All patients with heart failure should undergo HbA1c and fasting glucose testing. A diagnosis of diabetes mellitus is made when the HbA1c level is ≥ 6.5% (≥ 48 mmol/mol Hb) and fasting glucose is ≥ 126 mg/dl (≥ 7.0 mmol/l). For HbA1c levels between 5.7 and 6.5% (39–48 mmol/mol Hb) or fasting glucose levels between 100 and 125 mg/dl (5.6–6.9 mmol/l), an oral glucose tolerance test is recommended.

## Classification of Heart Failure

According to the current definition of the European Society of Cardiology [21••], heart failure can be classified into three groups:

- Heart failure with reduced ejection fraction (HFrEF)
- Heart failure with mildly reduced ejection fraction (HFmrEF)
- Heart failure with preserved ejection fraction (HFpEF)

Typical symptoms and signs of heart failure, along with the LVEF, are crucial for classifying the different types of heart failure. In the case of HFmrEF and HFpEF, an additional criterion such as elevated serum levels of natriuretic peptides, structural heart diseases, or diastolic dysfunction must be considered for diagnosis.

## Treatment of Patients with HFrEF and Diabetes

In general, the treatment for heart failure is similar for patients with and without diabetes mellitus. However, considering the significantly higher risk in patients with diabetes, the absolute risk reduction is often greater in these patients. The primary treatment approach for heart failure with reduced ejection fraction (LVEF ≤ 40%) is a four-drug regimen consisting of angiotensin-converting enzyme (ACE) inhibitors/angiotensin receptor-neprilysin inhibitor (ARNI), beta-blockers, mineralocorticoid receptor antagonist (MRA), and sodium-glucose co-transporter 2 (SGLT2) inhibitors [21••]. These four foundational therapies should be initiated early, and recent data suggest that an early up-titration within 6 weeks improves the prognosis compared to a standard approach [23].

### ACE Inhibitor/ARNI

ACE inhibitors were the first class of medications to demonstrate a reduction in mortality and morbidity in patients with heart failure with reduced ejection fraction [24, 25]. The beneficial effects of ACE inhibitors are observed in patients with and without diabetes [26–28]. Sacubitril/valsartan, an ARNI, has shown a significant reduction in cardiovascular death and heart failure–related hospitalization compared to ACE inhibitors [8]. The positive effects of sacubitril/valsartan are consistent across different HbA1c levels [9•]. Additionally, sacubitril/valsartan treatment has been associated with a slight decrease in HbA1c levels (− 0.14%) and a lower rate of initiating insulin therapy over a 3-year study period [29].

### Beta-Blockers

Beta-blockers have been shown to reduce mortality and morbidity in patients with heart failure with reduced ejection fraction who are already receiving ACE inhibitors and diuretics [30–33]. The benefits of beta-blockers extend to patients with heart failure and diabetes mellitus [34–37]. Beta-blockers have minimal effects on glucose metabolism in patients with diabetes, with non-selective beta-blockers showing a slight increase in fasting glucose levels [38].

### Mineralocorticoid Receptor Antagonist (MRA)

MRAs have consistently shown to reduce mortality and hospitalization due to heart failure in patients with and without diabetes [39, 40].

### Sodium-Glucose Co-Transporter-2 (SGLT2) Inhibitors

Two placebo-controlled studies were conducted to investigate the impact of SGLT2 inhibitors on patients with HFrEF, both with and without DM, who were already receiving optimal medical therapy (OMT). The DAPA-HF study enrolled patients with NYHA class II–IV and LVEF ≤ 40%, despite OMT, and elevated NT-proBNP levels. Patients with a GFR ≤ 30 ml/min/1.73 m^2^ were excluded from the study. Dapagliflozin demonstrated a 26% reduction in the relative risk of the primary endpoint, which combined worsened heart failure (hospitalization due to heart failure and the need for intravenous diuretic therapy) and cardiovascular death. Additionally, it led to an overall decrease in mortality [41]. These positive effects were observed in patients with and without DM, across the entire range of HbA1c levels, and were independent of concurrent antidiabetic treatment [41, 42]. The EMPEROR-Reduced trial included patients with HFrEF, both with and without diabetes mellitus, who had NYHA class II–IV, LVEF ≤ 40% despite OMT, and eGFR ≥ 20 ml/min/1.73 m^2^. Empagliflozin demonstrated a 25% reduction in the primary endpoint, which consisted of cardiovascular death and hospitalization due to heart failure. This reduction was primarily driven by a decrease in hospitalization rates [43]. The effect of empagliflozin on the primary endpoint remained consistent in patients with and without DM [44]. A meta-analysis of the DAPA-HF and EMPEROR-Reduced studies revealed a consistent impact of both medications on reducing hospitalizations due to heart failure or cardiovascular death, as well as overall mortality [45].

## Treatment of HFmrEF and HFpEF in Patients with Diabetes

For many years, no pharmacological therapy has conclusively shown a reduction in mortality and morbidity in patients with HFmrEF and HFpEF (LVEF > 40%). Retrospective data and subgroup analyses suggest that patients within the HFmrEF range (LVEF 41–49%) may benefit from similar therapeutic strategies as those with HFrEF [21••]. However, none of the studies encompassing patients with LVEF ≥ 40% (including HFmrEF and HFpEF) demonstrated a significant reduction in the primary endpoint. Diuretic therapy has been the sole option for alleviating symptoms.

However, the EMPEROR-Preserved study with empagliflozin and the DELIVER trials with dapagliflozin were the first trials in patients with an LVEF > 40% to demonstrate a significant reduction in the primary endpoint of cardiovascular death or hospitalization due to heart failure [46, 47]. A meta-analysis from EMPEROR-Preserved and DELIVER including 12,251 participants demonstrated that SGLT2 inhibitors, compared with placebo, reduced a composite of CV death and first hospitalization for HF by 20% (HR 0.80; 95% CI, 0.73–0.87), with consistent reductions in both components of the primary endpoint [48].

Given that individuals with diabetes mellitus often develop HFpEF, these findings suggest careful screening for HFpEF in patients with diabetes and giving empagliflozin or dapagliflozin as a therapeutic option for managing diabetes in confirmed HFpEF cases.

## Special Aspects

### Heart Failure in Diabetes and Chronic Kidney Disease (CKD)

In patients with type 2 diabetes mellitus (T2DM), CKD and HF represent the two most common first presentations of cardiovascular or renal disease. The presence of both comorbidities has a major impact on the prognosis. Epidemiological data suggest that patients with HFrEF who are at CKD stage 4 and 5 have a 50% survival probability over 20 months, in contrast to patients with HFrEF without CKD in which about 75% are still alive after 20 months [49]. The presence of DM in patients with CKD leads to an additional increase not only in CV mortality [50]. Thus, it is of utmost important to screen all patients with diabetes for the presence of CKD. Routine annual screening for CKD is recommended for all adults living with diabetes with spot urine sample UACR testing as well as serum creatinine testing to determine GFR [51, 52].

### Finerenone

Finerenone is a novel, selective, non-steroidal MRA that has been investigated in dedicated trials in patients with CKD and T2DM. Two clinical trials, FIGARO-DKD [53] and FIDELIO-DKD [54], investigated the impact of a novel non-steroidal mineralocorticoid receptor antagonist called finerenone on patients with both diabetes and CKD. Of note, patients with symptomatic HFrEF were excluded from these trials. The results of both trials demonstrated that compared to a placebo, finerenone reduced the risk of kidney failure and cardiovascular events. A comprehensive analysis of individual patient data from these trials, known as FIDELITY [55], included 13,171 patients. It revealed that when added to optimized renin-angiotensin system (RAS) blockade, finerenone significantly decreased the risk of a composite cardiovascular outcome, comprising cardiovascular death, non-fatal myocardial infarction, non-fatal stroke, or hospitalization due to heart failure, by 14%. The reduction in heart failure hospitalizations primarily contributed to this effect, even though patients with a history of heart failure were excluded from these studies. Moreover, the analysis indicated a non-significant trend towards a reduction in cardiovascular death or non-fatal myocardial infarction. Interestingly, a subset of patients in these trials were also treated with SGLT2 inhibitors. Predefined subgroup analyses suggested that the efficacy of finerenone is not dependent on concurrent SGLT2 inhibitor treatment [56].

In a phase 2 study called ARTS-HF [57], 1066 individuals with heart failure and type 2 diabetes mellitus and/or chronic kidney disease were randomized to receive various doses of finerenone with up-titration, or eplerenone with up-titration. The primary objective (percentage of participants with a > 30% decrease in plasma N-terminal pro–B-type natriuretic peptide (NT-proBNP) from baseline to day 90) was not statistically different between the eplerenone and finerenone groups, and the risk of hyperkalemia was also comparable. However, a significant secondary endpoint of all-cause death, cardiovascular hospitalizations, or worsening heart failure occurred less frequently in the finerenone 10 mg group compared to the eplerenone group (hazard ratio 0.56; *p* = 0.016). In addition, despite a trial duration of about 3 months, there were also fewer occurrences of all-cause death (*p* = 0.062) and cardiovascular death (*p* = 0.011) in the finerenone group compared to the eplerenone group [57]. The ongoing trial FINEARTS-HF trial (clinicaltrials.gov: NCT04435626) evaluates the efficacy and safety of finerenone in patients with symptomatic HF (NYHA class II II–IV and LVEF ≥ 40%) and will hopefully provide more information on the effect of finerenone in HF patients.

## Effect of Other Glucose-Lowering Drugs in Patients with Heart Failure

Beyond SGLT2 inhibitors, none of the other glucose-lowering drug classes has shown comparable effects in terms of heart failure. However, there are certain considerations and contraindications for the therapy of diabetes in patients with heart failure.

### Insulin

Current evidence suggests that insulin can be safely administered to patients with heart failure, and the use of insulin does not lead to an increased risk of heart failure hospitalization, as shown in the ORIGIN and the DEVOTE study [58, 59].

### Metformin

Metformin was long considered a first-line therapy for reducing blood glucose levels in patients with type 2 diabetes. Due to the potential risk of lactic acidosis in hemodynamically unstable patients, metformin was contraindicated in patients with heart failure for decades. However, various observational studies have shown that metformin may even lead to a reduction in mortality in patients with heart failure [60], so according to current understanding, metformin can be considered a safe drug for patients with diabetes and heart failure.

### Sulfonylureas

There is limited data regarding the effects of sulfonylureas on heart failure–associated outcomes. The recently published CAROLINA study, which compared the cardiovascular-safe DPP-4 inhibitor linagliptin [61] with the sulfonylurea glimepiride, showed that glimepiride does not increase the risk of hospitalization for heart failure [62]. Based on this data, modern sulfonylureas like glimepiride appear to be safe in patients with heart failure.

### Glitazones (Thiazolidinediones)

Glitazones increase insulin sensitivity and can lead to increased fluid retention in the kidneys. Clinical studies have shown an increased rate of heart failure hospitalizations with pioglitazone [63], making these substances contraindicated in patients with heart failure.

### DPP-4 Inhibitors

Four placebo-controlled cardiovascular outcome studies have investigated the effects of DPP-4 inhibitors in patients with diabetes and high cardiovascular risk. Only the SAVOR-TIMI 53 study showed an increased risk of hospitalization for heart failure with saxagliptin [64, 65]. This effect was not observed with sitagliptin or linagliptin in the TECOS [66, 67] and CARMELINA studies [61, 62], respectively. Alogliptin showed a non-significant trend towards increased hospitalization for heart failure in the EXAMINE study [68]. These data suggest that the increased risk of heart failure with saxagliptin is not a class effect but specific to this particular drug. Therefore, saxagliptin should not be used in patients with heart failure. None of the DPP-4 inhibitors has demonstrated a reduction in cardiovascular events compared to placebo.

### GLP-1 Receptor Agonists

Various large cardiovascular outcome studies have shown a reduction in cardiovascular endpoints with GLP-1 receptor agonists [69]. The reduction in cardiovascular endpoints in these studies was primarily driven by a reduction in atherosclerosis-related endpoints, and despite a moderate increase in heart rate by an average of 5–10 beats per minute, there was a neutral effect on the risk of hospitalization for heart failure for all these substances.

## Summary

**Fig. 1:**
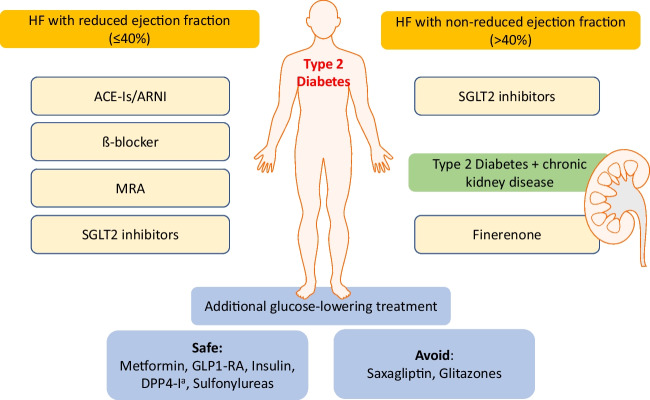
Medical treatment of patients with type 2 diabetes and heart failure with reduced or non-reduced ejection fraction. *ACE-I* angiotensin-converting enzyme inhibitor, *ARNI* angiotensin receptor/neprilysin inhibitor, *ß-blocker* beta-blocker, *MRA* mineralocorticoid receptor antagonist, *SGLT2* Sodium-glucose cotransporter-2, *GLP-1RA* Glucagon-like peptide-1 receptor agonists, *DDP4-I* Dipeptidyl peptidase 4 inhibitor

Given the increasing incidence of diabetes and heart failure as well as the impaired prognosis if both comorbidities come together, treatment of these patients requires an interdisciplinary, evidence-based approach to reduce morbidity and mortality in this high-risk population (Fig. 1).
